# *Piscirickettsia salmonis* elicited an antigen-specific cytotoxic response dependent on CD8+ T cells in Atlantic salmon

**DOI:** 10.3389/fimmu.2026.1803394

**Published:** 2026-04-01

**Authors:** Felipe Barraza-Rojas, Valentina Wong-Benito, Daniel Valdés-Henriquez, Kevin Maisey, Brian Dixon, Mónica Imarai

**Affiliations:** 1 Centro de Biotecnología Acuícola, Departamento de Biología, Facultad de Química y Biología, Universidad de Santiago de Chile, Santiago, Chile; 2 Department of Biology, University of Waterloo, Waterloo, ON, Canada; 3 Laboratorio de Microscopía Fotónica Avanzada, Departamento de Biología, Facultad de Química y Biología, Universidad de Santiago de Chile, Santiago, Chile; 4 Laboratorio de Inmunología Comparativa, Centro de Biotecnología Acuícola, Universidad de Santiago de Chile, Santiago, Chile

**Keywords:** antigen-specific cytotoxicity, Atlantic salmon, CD8+ T cell, cell-mediated immunity, cytotoxic T lymphocyte, *Piscirickettsia salmonis*, teleost immunity

## Abstract

*Piscirickettsia salmonis* is an intracellular bacterium responsible for Salmonid Rickettsial Septicemia (SRS) in Atlantic salmon. This pathogen survives within macrophages, hindering immune clearance. In mammals, CD8+ T lymphocytes represent a crucial component of adaptive immunity, as they specifically recognize and eliminate cells infected by intracellular pathogens. Evidence indicates that an analogous mechanism may also operate in teleost fish. Here, the role of CD8+ T lymphocytes in the immune response against *P. salmonis* was investigated in Atlantic salmon (*Salmo salar*). Using autologous dorsal fin-derived target cells and leukocytes from infected donors, an *ex vivo* model to assess antigen-specific cytotoxicity was established. Fin-derived cells internalized *P. salmonis* and trafficked the bacteria to lysosomal compartments, supporting antigen processing and MHC-I presentation. Sensitized leukocytes exhibited robust antigen-specific lysis of infected target cells, whereas non-specific lysis was negligible. Depletion of CD8a+ cells from sensitized peripheral blood leukocytes resulted in a loss of cytotoxic activity. These CD8a+ cells expressed CD3ζ and TCRβ transcripts, confirming the T-cell phenotype of the effector population. Together, these results provide the first functional evidence that Atlantic salmon mount CD8+ T cell-mediated cytotoxic responses against *P. salmonis*, highlighting cell-mediated immunity as a critical component of host defense, being a promising target for next-generation vaccines against SRS.

## 1 Introduction

Salmonid aquaculture in Chile experiences frequent infectious disease outbreaks that severely compromise fish welfare and cause substantial economic losses. Antibiotics and chemical treatments have proven helpful in controlling some bacterial diseases. Still, they have significant environmental impacts and limited effectiveness against bacteria that have developed mechanisms to evade the immune system and remain viable within target cells (1). One of the intracellular pathogens infecting salmonids and significantly affecting the aquaculture industry in Chile is *Piscirickettsia salmonis* (2). This bacterium infects Coho salmon (*Oncorhynchus kisutch*), Atlantic salmon (*Salmo salar*), and rainbow trout (*Oncorhynchus mykiss*) (3) causing the salmonid rickettsial septicemia (SRS). This disease has been reported on the Pacific and Atlantic coasts of the United States and Canada, as well as in Ireland, Scotland, Norway, and Tasmania (4–7). *P. salmonis* is a Gram-negative, non-motile, non-encapsulated, facultative intracellular, pleomorphic bacterium with a coccoid shape measuring between 0.5 and 1.5 µm in diameter. Molecular phylogenetic analyses based on 16S rRNA gene sequencing classified *P. salmonis* as a proteobacterium of the class Piscirickettsiae (5, 8). The infection affects the kidneys, liver, spleen, gut, brain, and gills, resulting in fish mortality. *P. salmonis* can localize in the cytoplasmic vacuoles of myeloid cells, such as macrophages and monocytes, allowing it to survive and replicate, which is a strategy used to evade the host’s immune response (9, 10).

To date, the mortality rate caused by this pathogen has not been reduced and it remains a persistent problem in the salmon industry (2). Vaccination is the main prophylactic measure for disease control in modern aquaculture; however, the available vaccines and adjuvants are not sufficiently effective to control SRS (11–13). In this regard, our laboratory seeks to identify and characterize the immune response that could protect salmonid fish against *P. salmonis*, to guide the development of improved vaccines against SRS. One understudied aspect of fish immunity against *P. salmonis* that could sustain protection is the mechanisms of immunity mediated by cytotoxic T lymphocytes (CTL).

T cell-mediated cytotoxicity is an essential component of the effector mechanisms against cells infected by intracellular microorganisms, such as bacteria and viruses. Recent advances have expanded the understanding of how this cytotoxic response operates in teleost fish, particularly regarding CD8+ T cell development, activation, and effector functions (14, 15). In mammals, cytotoxic T cells display a CD8 coreceptor on their surface, either as a homodimer composed of two α chains (CD8αα) or as a heterodimer consisting of one α and one β chain (CD8αβ). The CD8 molecules are used as a marker for cytotoxic T cells. CD8+ T cells recognize cognate peptides bound to the major histocompatibility complex class I (MHC-I) molecules on the surface of target cells, inducing cell death (16, 17). The CD8α and CD8β genes have been reported in fish species such as rainbow trout, ginbuna carp (*Carassius auratus*), and orange-spotted grouper (*Epinephelus coioides*) (18–22), and the presence of CD8+ T lymphocytes has been confirmed in some teleost fish using species-specific antibodies (14, 18, 23).

CTL activity has been demonstrated in only a few fish species, including ginbuna crucian carp (*Carassius langsdorfii*), rainbow trout and orange-spotted grouper (*Epinephelus coioides*). In ginbuna crucian carp, intraperitoneally infected with hematopoietic necrosis virus (CHNV), the virus-specific cytotoxic activity was demonstrated for the first time (24, 25). Moreover, the virus-specific cytotoxic response was transferable, as recipients of leukocytes from immune syngeneic donors were protected from CHNV infection. Virus-specific cytotoxic T cells were found in a mixed leukocyte culture (MLC) analysis, also in ginbuna crucian carp (26). In this assay, lymphocytes from fish infected with CHNV proliferate *in vitro* upon stimulation with virus-infected syngeneic target cells, and the effector cells successfully lyse CHNV-infected syngeneic target cells after several days (26). Furthermore, using a CD8α-specific monoclonal antibody they demonstrated that the antiviral cytotoxic cells were CD8+ (27). In another study, homozygous rainbow trout (clone C25) and isogenic target cells (cell line RTG-2) expressing matched-MHC class I alleles of the locus Onmy-UB (28) were used in an infection trial. Peripheral blood leucocytes (PBL) from rainbow trout infected with the viral hemorrhagic septicaemia virus (VHSV) or immunized with DNA encoding the virus G protein (28, 29) successfully lysed VHSV-infected RTG-2 cells but not the control uninfected cells. In addition, leucocytes isolated from virus-infected fish showed a higher transcriptional level of the CD8α gene when compared to PBL from uninfected control fish, suggesting that the cytotoxic activity was due to CD8+ cells. Finally, also in orange-spotted grouper (*Epinephelus coioides*), sensitized CD8α+ PBL have antigen-specific cytotoxic activity against nervous necrosis virus (NNV) in a class I MHC restricted manner (18).

In Atlantic salmon, the sequences of the CD8α and CD8β gene chains have been characterized, and their gene expression has been observed in major lymphoid organs such as the thymus, anterior kidney, and spleen, as well as in mucosa-associated organs such as the intestine (30). The role of lymphocytes in Atlantic salmon has been examined in relation to infections caused by Piscine orthoreovirus (PRV-1), which leads to heart inflammation (31). *In situ* hybridization and RT-PCR analysis, combined with phase contrast microscopy, revealed that leukocytes in the hearts of infected fish co-localized with PRV-1, CD8+ cells, MHC-I, and granzyme signals. PRV levels were inversely correlated with the presence of CD8+ cells and MHC-I expression. This led to the hypothesis that CTLs could play a role in the pathogenesis of this infection (31). Recently, CD8+ lymphoid cells have been detected in peripheral blood cells of PRV+ and PRV- salmon (32) but functional studies on CTLs or specific antigen recognition have not been conducted.

The role of CD8+ T cells in protecting against intracellular bacteria has been studied in mammals. Bacteria such as *Listeria monocytogenes* that escape into the host cell cytoplasm can deliver antigens into the MHC I pathway, resulting in strong activation of CD8+ cytotoxic T lymphocytes (CTLs) (33). These CTLs are essential for resolving the infection. On the other hand, intravacuolar pathogens such as Mycobacteria and Nocardia block the fusion of phagosomes and lysosomes and reduce the acidification inside vacuoles. This limits the direct presentation of antigens through MHC I. Despite this, CD8+ CTLs still play an important role in protection by recognizing antigens from infected cells or through a process called cross-presentation (34, 35). They work alongside CD4+ T cells to enhance the immune response.

To the best of our knowledge, there are no reports of the role of CTL against bacteria in Atlantic salmon. In this context, we aim to investigate whether salmon elicit an immune response against *P. salmonis*, mediated by CD8+ cytotoxic T cells. Understanding the cellular mechanisms of the immune response and potential protection against *P. salmonis* can aid in developing new and more effective therapeutic strategies for Salmonid Rickettsial Septicemia, including improved vaccines.

## 2 Methods

### 2.1 Fish

Atlantic salmon (*Salmo salar*) of approximately 80 g were obtained from Salmones Blumar S.A. and maintained at the Experimental Fish Facility at the Center for Aquaculture Biotechnology, in freshwater at a biomass of approximately 6 kg/m^3^, 11-12 °C, and continuous aeration. Fish were fed with commercial pellets (Golden Optima, Biomar, Chile) twice a day until satiety. When corresponding, fish were acclimated for two weeks before treatments. During the experiments, the pH (7–7.5), dissolved oxygen (8.9–9.5 mg O_2_/L), and ammonia (<0.1 mg/L) were recorded daily.

### 2.2 Primary cultures of dorsal fin cells

Dorsal fin cells from Atlantic salmon were established following the protocols described by Zhou et al. (36). Dorsal fins were aseptically excised from pre-smolt Atlantic salmon (average weight 80 g), previously euthanized with benzocaine. The fins were washed three times with phosphate-buffered saline (PBS), maintained in cold fetal bovine serum (FBS), and cut into 1 mm fragments. The tissue fragments were placed into six-well culture plates and incubated at 18 °C. The culture medium consisted of L-15 medium (Cytiva) supplemented with 40 μM 2-mercaptoethanol (Gibco), 50 μg/mL gentamicin (Gibco), 5 mM HEPES (Corning), 5 mM non-essential amino acids (Corning), and 20% v/v FBS (Hyclone). Once cells reached approximately 80% confluence, the culture medium was gently removed, and the cells were washed twice with phosphate-buffered saline (PBS, pH 7.4) to eliminate residual serum and cell debris. Cells were detached using 0,1% trypsin (0,25% trypsin in PBS, GIBCO Thermo Fisher Scientific) and incubated at 18 °C for 5–7 minutes until most cells detached. The enzymatic reaction was stopped by adding an equal volume of L-15 medium supplemented with 10% FBS. The cell suspension from each well was collected individually, transferred to sterile 15 mL conical tubes, and centrifuged at 300 x g for 5 minutes at 4 °C. The supernatant was discarded, and the pellet was resuspended in 1 mL of cold PBS containing 2% FBS for further analysis.

### 2.3 SHK-1 culture

SHK-1 cell line (ECACC 97111106) obtained from salmon head kidney (37) was grown in T-75 flasks at 18 °C in Leibovitz’s 15 medium supplemented with 10% FBS, 4 mM L-glutamine and 40 μM 2-mercaptoethanol. Cells were propagated by washing twice with PBS and adding 0,1% trypsin to detach the cells. Trypsin was removed at each subculture by centrifuging the harvested cells at 100 x g for 5 minutes, then resuspending the pellet in fresh medium.

### 2.4 Confocal microscopy and Flow cytometry analysis of dorsal fin cells

For confocal microscopy analysis, cultured cells were detached with 0,1% trypsin, counted in a Neubauer chamber after trypan blue exclusion, and 1 × 10^5^ cells were seeded onto poly-L-lysine-coated coverslips. After 24 h of adherence, cells were washed twice with PBS, then fixed with 2% paraformaldehyde for 15 minutes at room temperature. Blocking was done with 1% BSA in PBS for 30 minutes, followed by incubation with primary antibodies: rabbit polyclonal anti-MHC-I (1:50) (38), mouse polyclonal anti-CD80/86 (1:100) (39), and rabbit polyclonal anti-CD4-1 (1:50) (40) for 1 hour at room temperature. After washing, secondary antibodies Alexa Fluor 647 donkey anti-rabbit IgG (H+L) (1:800) or Alexa Fluor 488 donkey anti-mouse IgG (H+L) (1:600) were applied for 45 minutes in the dark at room temperature. Nuclei were counterstained with DAPI (1µg/mL) for 10 minutes. Coverslips were mounted using a DABCO solution (2,5% 1,4-diazabicyclo [2.2.2] octane in 90% glycerol, 10% PBS). Imaging was acquired with a Zeiss confocal microscope LSM-800, and processed using Zeiss ZEN software (version 3.5, Blue edition). For Flow cytometry analysis, fin-derived cells (5x10^5^ per sample) were incubated in PBS supplemented with 2% FBS (IF medium) for 30 minutes at 4 °C before staining. After centrifugation at 250 g for 5 minutes, cells were resuspended in 200 μL IF medium containing the primary antibody rabbit polyclonal anti-MHC-I (1:50) (38), mouse polyclonal anti-CD80/86 (1:100) (39), or the antibody rabbit polyclonal anti-CD4-1 (40). The anti-CD4–1 antibody was titrated using 5x10^5^ cells, and the optimal working dilution determined for subsequent experiments was 1:50. Cells were incubated for 45 minutes at 4 °C, then were washed with 600 μl IF medium. After washing, samples were incubated with 300 μL IF containing the secondary antibody. Secondary antibodies used were Alexa Fluor 647 donkey anti-rabbit IgG (H+L) (1:800) (Life Technologies) and Alexa Fluor 647 donkey anti-mouse IgG (H+L) (1:600) (Life Technologies). Channel emissions were allophycocyanin (APC) 660–680 nm. Cells were incubated for 30 minutes at 4 °C, then washed with 800 μL IF media and resuspended in 400 mL IF media prior to analysis. Unstained cells were used to assess autofluorescence, and samples incubated only with secondary antibodies served as isotype controls. Prior to acquisition, cells were incubated with 5 μg/mL propidium iodide (PI) for exclusion of non-viable cells and staining was detected in the PE-channel. Data were acquired on a BD FACSCanto II flow cytometer (BD Biosciences). A minimum of 10,000 events per sample was acquired using FACSDiva software (BD Biosciences). Two populations were consistently observed on FSC-A/SSC-A plots: a smaller, low-granularity population (FSC^low^SSC^low^) and a larger, more granular population (FSC^hi^SSC^hi^). Both populations were analyzed by applying gates specific to each group. Debris was excluded based on FSC-A versus SSC-A, singlets were identified using FSC-A versus FSC-H, and live PI-negative cells were selected for downstream analysis. All data analyses were performed on live cells using FlowJo 10.1.

### 2.5 RNA extraction and synthesis of cDNA

Fin cells from culture were homogenized in 1 mL TRIzol Reagent (Invitrogen, Thermo Fisher Scientific) using a tissue cell disruptor (Omni International). Total RNA was extracted according to the manufacturer´s protocol. Briefly, the extracted RNA was suspended in diethyl pyrocarbonate–treated water (Invitrogen) and quantified. RNA (1.5 µg) samples were treated with RQ1 RNase-free DNase (Promega), and cDNA synthesis was performed using reverse transcriptase Moloney murine leukemia virus (Promega) and oligo (dT) (Promega) according to the manufacturer’s instructions. RNA samples were stored at -80 °C, and cDNA at -20 °C, until use.

### 2.6 RT-PCR analysis

PCR was performed using GoTaq polymerase (Promega). Target genes and primer sequences are listed in **Table 1**. PCR reactions were performed using 25 µL containing 12.5 µL 5X Green master mix GoTaq reaction buffer (Promega), 6,5 µL ultrapure distilled water (Invitrogen), 2 µL 5 pM forward primer, 2 µL 5 pM reverse primer, and 2 µL cDNA template. Amplification was done as follows: 5 min denaturation at 95 °C followed by 35 PCR cycles of 45 s at 95 °C, 45 s at 60 °C, and 45 s at 72 °C. Final extension was carried out for 5 min at 72 °C. Amplicons were loaded onto 2% agarose gels and were separated by electrophoresis. PCR products were stained with ethidium bromide and visualized under UV light.

**Table 1:** Sequence of primers.

Gene	Accession No.	Sequence (5’→ 3’)
MHC-I	XM_045709561.1	F 5’ CCA GTG ACC TGC CAC GCG AC 3’ R 5’ TGT TGG CCG GAA CAA AGC CT 3’
β−Actina	NM_001123525.1	F 5’ GAC GAG GCT CAG AGC AAG AG 3’ R 5’ GTT GGC TTT GGG GTT GAG TG 3’
16S rRNA	MN023082.1	F 5’AGG GAG ACT GCC GCT GAT A 3’ R 5’ ACT ACG AGG CGC TTT CTC A 3’
CD8α	NM_001123583.1	F 5’ CAC TGA GAG AGA CGG AAG ACG 3’ R 5’ TTC AAA AAC CTG CCA TAA AGC 3’
CD3ζ	NM_001123620.1	F 5’ TGT TTG CAG TTG TGT TAG TCC C 3’ R 5’ GGG AGA AGA GAC GAG AGC TAA AG 3’
TCRβ	XM_045706178.1	F 5’ CTA GTG TGT GTA GCC ACC CG 3’ R 5’ TCT CTC CCC CAC TTT GAC CT 3’

### 2.7 P. salmonis

The strain LF-89 of *P. salmonis* (ATCC VR-1361) was cultured at 18 °C in liquid medium formulation 5 (patent pending) (41) with agitation at 100 rpm approximately for five days or until the optical density reach at 600 nm (OD_600_ nm). Subsequently, the viable bacteria population was quantified using LIVE/DEAD BacLight Bacteria viability and counting KIT (molecular Probes) on a BD FACSCanto II Flow Cytometry system (BD Biosciences). Live bacteria were used for *in vitro* cell infections. For inactivation, 1 mL of culture was centrifuged at 10,000 g for 3 minutes at 18 °C. The bacterial pellet was resuspended in PBS and heat-inactivated at 100 °C for 30 minutes (42).

### 2.8 Detection of *P. salmonis* by PCR

The 16S rRNA gene (**Table 1**) was amplified following the protocol described by Kataras et al. (43) to detect the presence of *P. salmonis* in bacterial cultures. Genomic DNA was isolated from 1 mL of *P. salmonis* culture by centrifugation at 10,000 x g for 3 minutes at 18 °C. The resulting pellet was processed using the Wizard Genomic DNA Purification Kit (Promega, WI, USA) according to the manufacturer’s protocol to obtain high-purity DNA suitable for downstream molecular analysis. PCR reactions were then performed to evaluate 16S gene expression using the SSoAdvanced Universal SYBR green kit (Biorad) on an AriaMx real-time PCR system (Agilent Technologies). Finally, the PCR product size was verified by visualization of a band on a 2% agarose gel.

### 2.9 Infection in dorsal fin cells

Primary dorsal fin cells from Atlantic salmon were cultured in six-well plates and maintained at 18 °C in supplemented L-15 medium as mentioned previously. For Infection assays, *P. salmonis* (LF-89 strain) was labeled with SYTO 9 (Invitrogen), a green, fluorescent nucleic acid stain, following the manufacturer’s instructions. Briefly, bacterial suspensions were incubated with SYTO 9 (5 μM final concentration) for 15 minutes in the dark at room temperature, followed by two washes with sterile phosphate-buffered saline (PBS, pH 7.4) to remove excess dye (**Supplementary Figure 1**). Labeled bacteria. were used immediately to infect dorsal fin cells at a multiplicity of infection (MOI) of 10. Infected cultures were incubated at 18 °C for 24 hours to allow bacterial internalization. To visualize the uptake and intracellular localization of the bacteria, the infected cells were washed twice with PBS and labeled for lysosomal compartments by incubating them with Lysotracker Deep Red (Invitrogen) for 45 minutes at 18 °C in the dark. Then, the cells were fixed with 2% paraformaldehyde in PBS for 10 minutes at room temperature. After this, the wells were washed three times with PBS, and nuclei were stained with DAPI (4’,6-diamidino-2-phenylindole; Invitrogen, 1ug/mL) for 10 minutes in the dark. Images were acquired using a Zeiss LSM 800 confocal laser scanning microscope (Carl Zeiss, Germany) and then processed and analyzed using ZEN blue 3.5 software (Carl Zeiss). Colocalization of *P. salmonis* with lysosomal compartments was evaluated by overlaying the green (bacteria) and red (lysosomes). Controls cells were processed in parallel using the same workflow.

### 2.10 Peripheral blood leukocytes preparation

PBL were isolated using a hypotonic lysis procedure to remove erythrocytes while preserving leukocyte viability and functionality (44). Blood was collected from the caudal vein with a 1 mL syringe (Nipro). Erythrocytes were lysed by mixing 1 mL of blood with 8 mL of ice-cold deionized water, followed by inversion for 20 seconds. Subsequently, 1 mL of 10X PBS was added to restore isotonicity. The PBL suspension was placed on ice for 5 to 10 minutes to facilitate clumping and sedimentation of cell debris. The suspension was filtered through a 70 μm cell strainer (BD Falcon) and washed by centrifugation at 400 g for 5 minutes using L-15 medium. All procedures were performed at 4 °C. Cell counts and viability assessments were routinely conducted using trypan blue.

### 2.11 Cytotoxic activity assay

**Figure 1:**
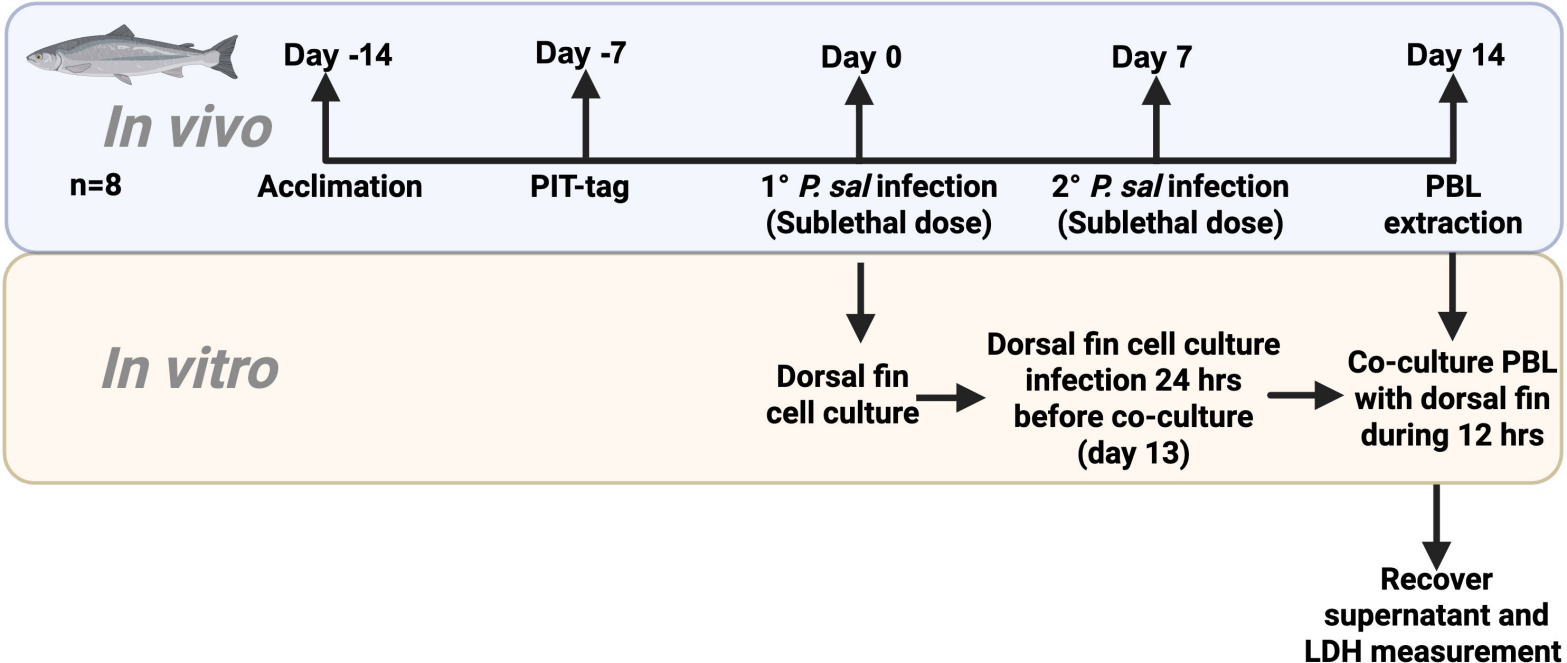
Schematic representation of the experimental strategy for the ex vivo cytotoxicity assay in Atlantic salmon using an isogenic framework. Following a 7-day acclimation period, fish were subjected to passive integrated transponder (PIT) tagging. Seven days later, dorsal fin explants were collected for primary cell culture. Intraperitoneal injections of a sublethal dose (5x10^6^) live *Piscirickettsia salmonis* were administered on days 0 and 7 to induce CD8+ T-cell activation. On day 13, dorsal fin cultures were exposed *in vitro* to *P. salmonis* at a multiplicity of infection (MOI) of 10. Peripheral blood leukocytes were then isolated from each fish and co-cultured with the corresponding autologous fin cells for 12 hours. Cytotoxicity was determined by quantifying lactate dehydrogenase (LDH) release in culture supernatants (SN).

To assess isogenic antigen-dependent T cell cytotoxicity assays, 8–14 days cultures of dorsal fin cells obtained from PIT tagged fish (∼80 g) were prepared and identified with the PIT tag code of the donor fish (**Figure 1**). Then, the PIT-tagged fish were first clipped on the dorsal fin for the primary culture and then intraperitoneally infected (days 0 and 7) with 200 μL of suspension in physiological saline containing sublethal doses of *P. salmonis* (5x10^6^ bacteria/mL per fish) (**Figure 1**). Sensitized leucocytes (Effector cells, E) were obtained on day 14 from the peripheral blood of each PIT-tagged infected fish. Twenty-four hours before the beginning of the cytotoxicity assay, the dorsal fin cells were exposed to live or heat-inactivated *P. salmonis* (MOI 10) and incubated for 24 h at 18 °C in infected medium (L-15 supplemented with 10% FBS) (**Figure 1**) to allow processing of bacterial proteins and peptides to be presented on MHC molecules. Then, the medium was replaced with L-15 supplemented with 10% FBS, and *P. salmonis*-sensitized PBL (E) were co-cultured with dorsal fin cells (T) at an E:T ratio of 10:1 for the indicated time at 18 °C or 37 °C. Supernatants from the culture were collected for further analysis. Several control groups were used to analyze the antigen presentation: fin cells alone (group l: spontaneous lysis), fin cells exposed to live or heat-killed *P. salmonis* (group 2: control bacterial-induced lysis), fin cells co-cultured with sensitized PBL (group 3: non-specific lysis), fin cells exposed to live or heat-killed *P. salmonis* and co-cultured with sensitized PBL (group 4: specific lysis), and fin cells incubated with lysis buffer (group 5: 100% lysis), for the cytotoxic assay, cell culture media were collected and centrifuged at 400 x g for 5 minutes at 4 °C.

### 2.12 LDH cytotoxicity assay

Supernatants were analyzed for lactate dehydrogenase (LDH) using the CyQuant™ LDH Cytotoxicity Assay (Thermo Fisher #C20301) (45). For the assay, 50 μl of each sample was analyzed in triplicate in a 96-well plate. Wells containing medium alone served as negative controls, and the kit-supplied reagent served as the positive control. To establish a 100% lysis reference, fin cells were treated with the provided lysis buffer for 45 minutes at 18 °C. The media from these cells were then centrifuged under the same conditions, and 50 μL of the resulting supernatant was added to the plate. After a 30-minute incubation at 37 °C, absorbance was measured at 490 nm and 680 nm. The percentage of cytotoxic activity was calculated using the formula below:


$$\%\;of\;cytotoxicity=100\text{ x}\frac{\left(\text{LDH release in Experimental}-\text{Spontaneous LDH release}\right)}{\left(\text{Total LDH release}-\text{Spontaneous LDH release}\right)}$$

### 2.13 Fluorescence-activated cell sorter

For cell sorting, isolated cells from PBL were centrifuged at 250 g for 5 minutes and resuspended in 400 μL of undiluted hybridoma supernatant of the mouse monoclonal anti-salmon CD8α (Mab 6A8) produced against the Atlantic salmon recombinant CD8α (21). Cells were incubated for 45 minutes at 4 °C and then washed with 1 mL of IF medium. After washing, samples were incubated with 400 μL IF containing the secondary antibody Alexa Fluor 488 donkey anti-mouse IgG (H+L) (Life Technologies) (1:600) for 30 minutes at 4 °C. Stained cells were washed with IF medium and resuspended in 500 µL IF medium for acquisition on a FACSMelody cell sorter (Becton Dickinson Biosciences). At least 30,000 events per sample were recorded. CD8 staining was detected in the FITC channel. Flow cytometry analyses always included incubation with 5 μg/mL PI for cell viability staining and exclusion of dead cells. The PI staining was detected in the PE channel. Leucocytes exhibited a characteristic distribution in forward (FSC) and side scatter (SSC), allowing the distinction between the lymphoid (FSC^low^SSC^low^) and the myeloid cell population (FSC^hi^SSC^hi^). Cells were analyzed on a gate set for lymphocyte-sized cells. Autofluorescence measurement and isotype control were always included. Debris was excluded based on FSC-A versus SSC-A, singlets were identified using FSC-A versus FSC-H, and PI-negative cells were selected for downstream analysis. Cells were then sorted into CD8^-^ (CD8-negative) and CD8^+^ (CD8-Positive) fractions. Sorted populations were collected cells into tubes containing heat-inactivated FCS and were analyzed (post-sorting) for further assays. All data analyses were performed on live cells using FlowJo 10.1.

## 3 Results

### 3.1 Target cells derived from the dorsal fin of Atlantic salmon

**Figure 2:**
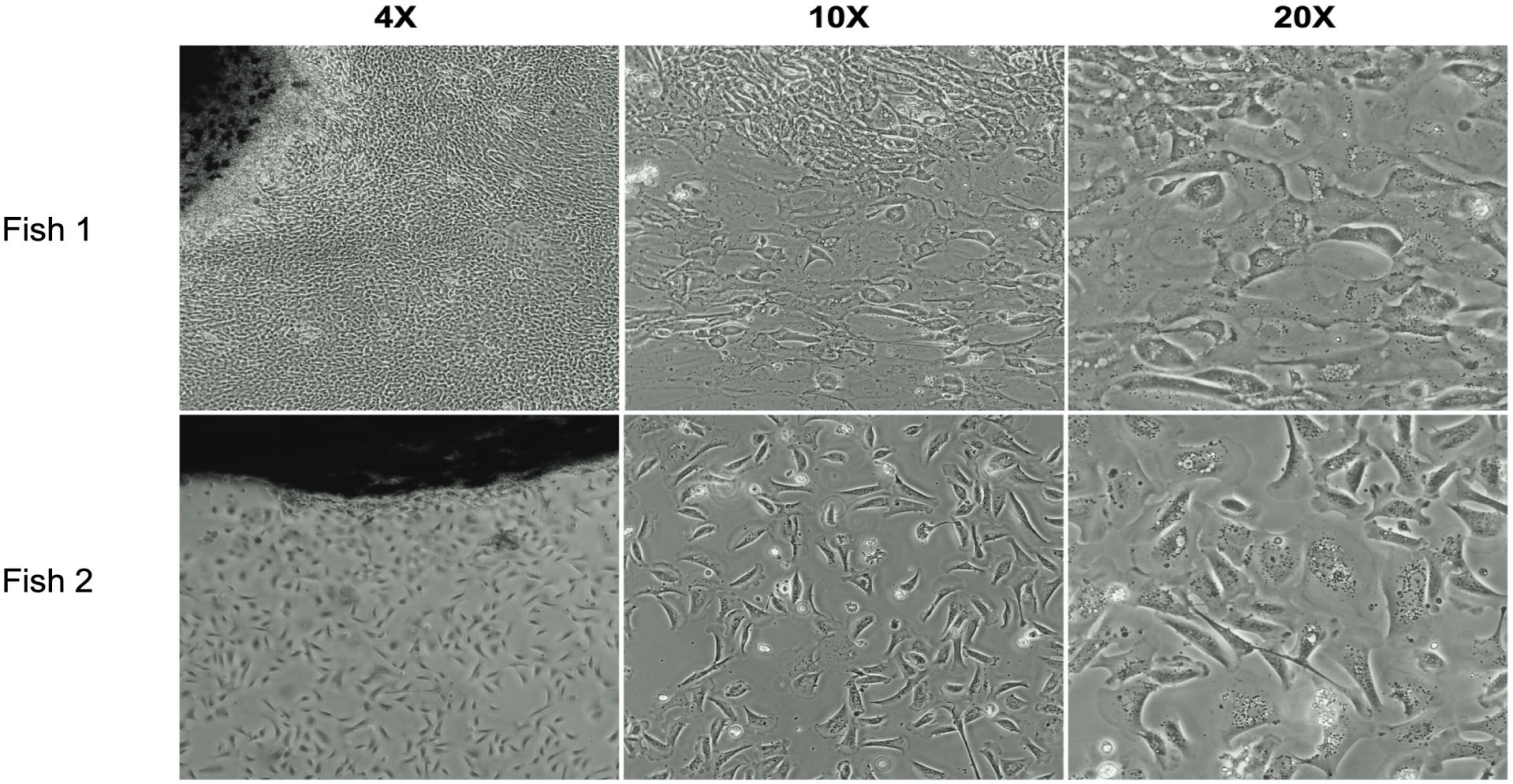
Target cells derived from the dorsal fin of Atlantic salmon. Optical microscopy images show primary cell cultures from the dorsal fin after 7 days of incubation. The cultures exhibit a heterogeneous population consisting of small, rounded epithelial-like cells and larger, elongated fibroblast-like cells distributed across the culture surface. Fin cell cultures of 2 fish are shown. Upper figures (Fish 1). Lower panel (Fish 2).

**Figure 3:**
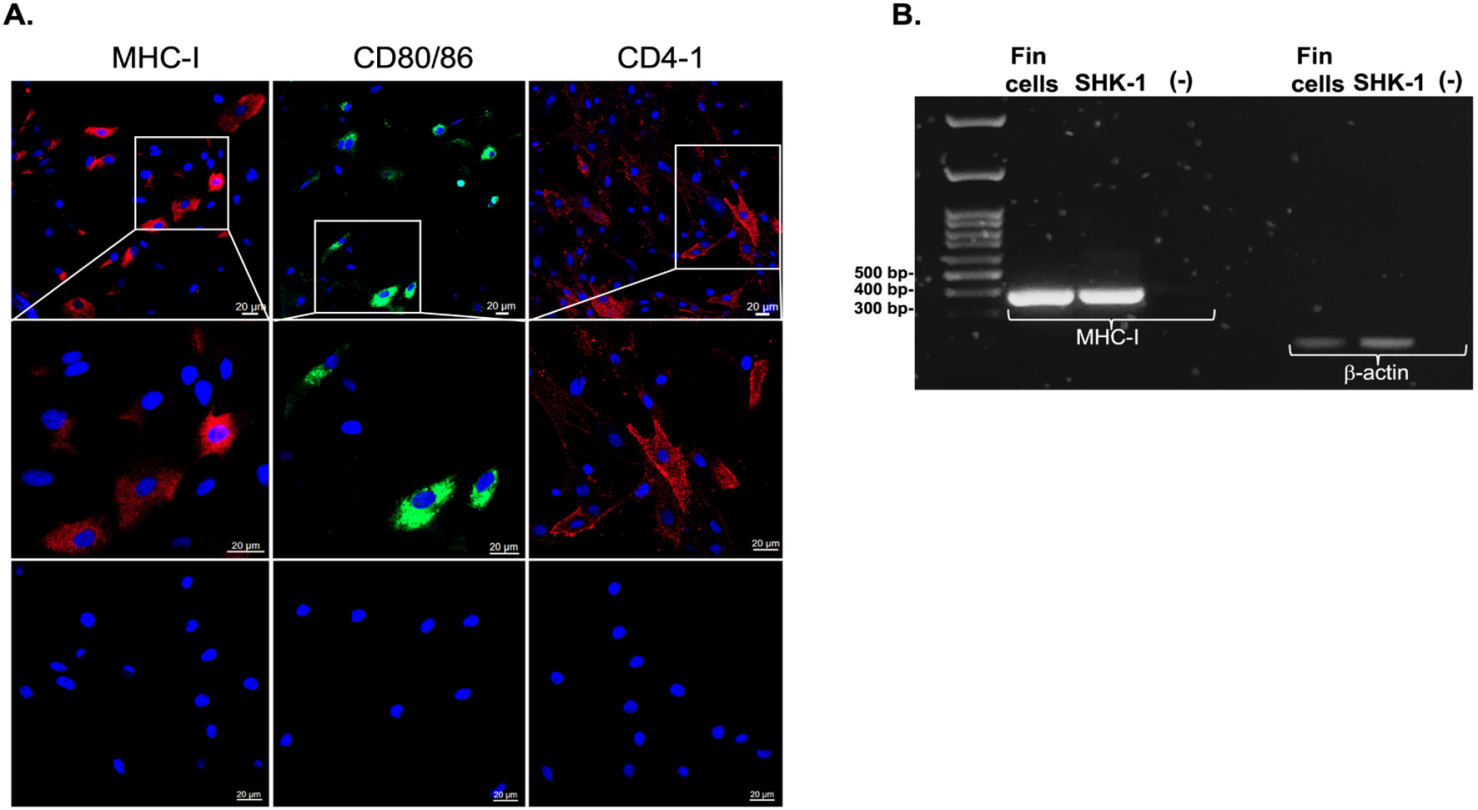
Detection of MHC-I, CD80/86, and CD4–1 in fin-derived cells analyzed by confocal microscopy and detection of MHC-I gene expression by conventional PCR. **(A)** Confocal immunofluorescence images showing the presence of MHC-I (red), CD80/86 (green), and CD4-1 **(red)** in fin-derived cells. Nuclei were stained with DAPI (blue). The upper panels show general views; the middle panels show higher magnification of representative cells of the upper panel. Lower panel are the autofluorescence controls, with cell incubation conducted with the secondary antibodies only. Images were acquired using alpha Plan-Apochromat 63x/1.46 Oil M27. **(B)** RT-PCR products were separated using a 1% agarose gel electrophoresis, which revealed a distinct ~350 bp band consisting with MHC−I. Amplification of β−actin was used as an internal control, while cDNA from SHK−1 cells, a macrophage−like Atlantic salmon line known to express MHC−I, served as the positive control.

**Figure 4:**
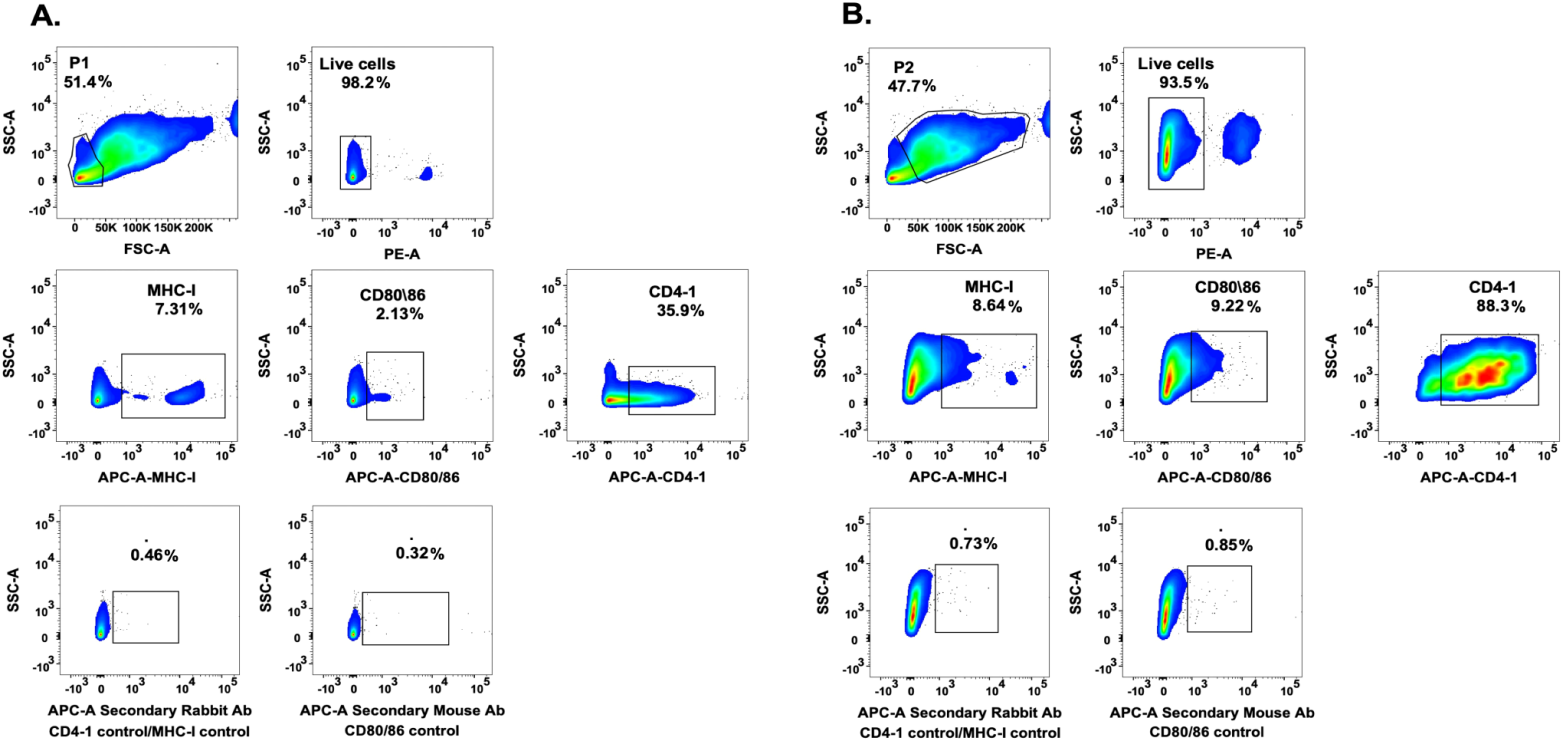
Characterization of primary cell cultures derived from the dorsal fin of Atlantic salmon by flow cytometry. **(A)** The upper panel presents the P1 cell population, defined by FSC-A versus SSC-A gating and cell viability assessed using PE-A emission. The middle panel displays the percentage of MHC−I-, CD80/86-, and CD4-1-positive cells within P1. The lower panel presents the autofluorescence controls. **(B)** The upper panel presents the P2 cell population, defined by FSC-A versus SSC-A gating and cell viability measured using PE-A emission. The middle panel shows the percentage of MHC−I-, CD80/86-, and CD4-1-positive cells within P2. The lower panel displays the autofluorescence controls.

To analyze antigen-dependent cytotoxicity, adherent cells from the dorsal fins of tagged fish were prepared to use as targets. Optical microscopy images of 7-day cultures showed heterogeneous morphology, characterized by the coexistence of small, rounded cells and larger, elongated fibroblast-like cells distributed throughout the culture surface (**Figure 2**). Such heterogeneity was consistently observed in independent cultures (n=11 fish) and reflects the mixed cellular composition typical of fin-derived explants (**Supplementary Figure 2**). Fin cells were in addition stained with antibodies targeting key molecules for antigen presentation and T cells activation, specifically salmonid MHC class I (UCA), CD80/86, and CD4–1 and subsequently examined using confocal microscopy. The analysis showed positive staining with all three antibodies (**Figure 3A**), while the experimental negative controls showed no fluorescence staining (**Figure 3A**). Other two biological replicates are shown in **Supplementary Figure 3**. Conventional PCR analysis further confirmed the expression of the *mhc-i* in fin cells derived from the dorsal fin (**Figure 3B**). To verify the presence of the molecules on the cell surface of fin cells, flow cytometric analysis of fin cells was performed in one of the cultures. Two distinct cell populations based on their forward scatter (FSC) and side scatter (SSC) profiles were identified (**Figure 4**). In this sample, one population, including approximately half of the cells, exhibited lower FSC and SSC values (FSC^low^SSC^low^), consistent with smaller, less granular epithelial-like cells observed under light microscopy (**Figure 4A**). The second population, which accounted for the other half of the cultured fin adherent cells, displayed higher FSC and SSC values (FSC^high^SSC^high^), indicative of larger, more complex cells (**Figure 4B**). Immunofluorescence analysis using antibodies against MHC class I (UCA), CD80/86, and CD4–1 detected the molecules on the alive cells (**Figure 4**), which is consistent with the analysis performed using confocal microscopy (**Figure 3**). In addition, labelling showed that approximately 7% of the FSC^low^SSC^low^ population expresses MHC class I molecules, 2% exhibit CD80/86, and 36% are CD4-1-positive cells (**Figure 4A**). In the FSC^high^SSC^high^ cells, 8% contain MHC class I molecules, 9% are CD80/86-positive cells, and 88% exhibit CD4–1 molecules (**Figure 4B**).

### 3.2 Evidence of bacterial internalization in dorsal fin cells of Atlantic salmon

**Figure 5:**
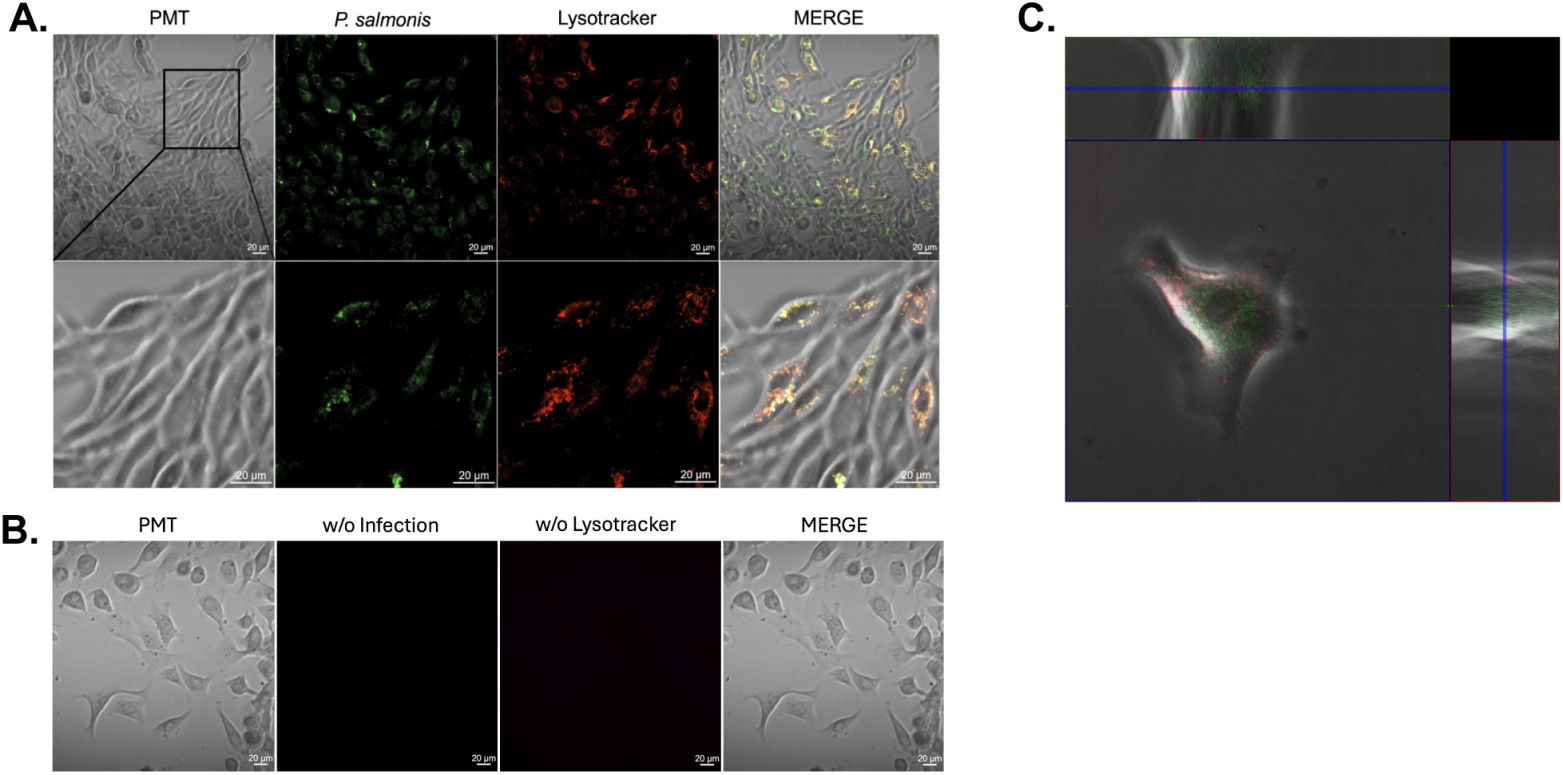
Confocal microscopy analysis of dorsal fin-derived cells from Atlantic salmon infected with *P. salmonis*. **(A)** Primary dorsal fin cells were infected for 24 hours with SYTO 9-labeled *P. salmonis* (green) and stained with Lysotracker Red to visualize lysosomal compartments. Merged images are colocalization (yellow/orange signal) between bacterial and lysosomal fluorescence. Each row shows split images of the same microscopic field. Images were acquired using an LD A-Plan 40X/0.55 Ph1 objective. **(B)** Control condition corresponding to **(A)**, showing one uninfected primary fin-derived cells processed under identical staining and imaging conditions. Each row shows split images of the same microscopic field. Images from the same microscope field were acquired using an LD A-Plan 40X/0.55 Ph1 objective. **(C)** The central panel displays an XY overlay of *Piscirickettsia salmonis* (green) and Lysotracker (red) positive compartments in a fin-derived cell. The upper panel presents the orthogonal YZ view, illustrating the intracellular localization of *P. salmonis* within Lysotracker-positive compartments. The right panel shows the orthogonal XZ view, confirming colocalization in three dimensions. Images were acquired using an LD A-Plan 40X/0.55 Ph1 objective.

**Figure 6:**
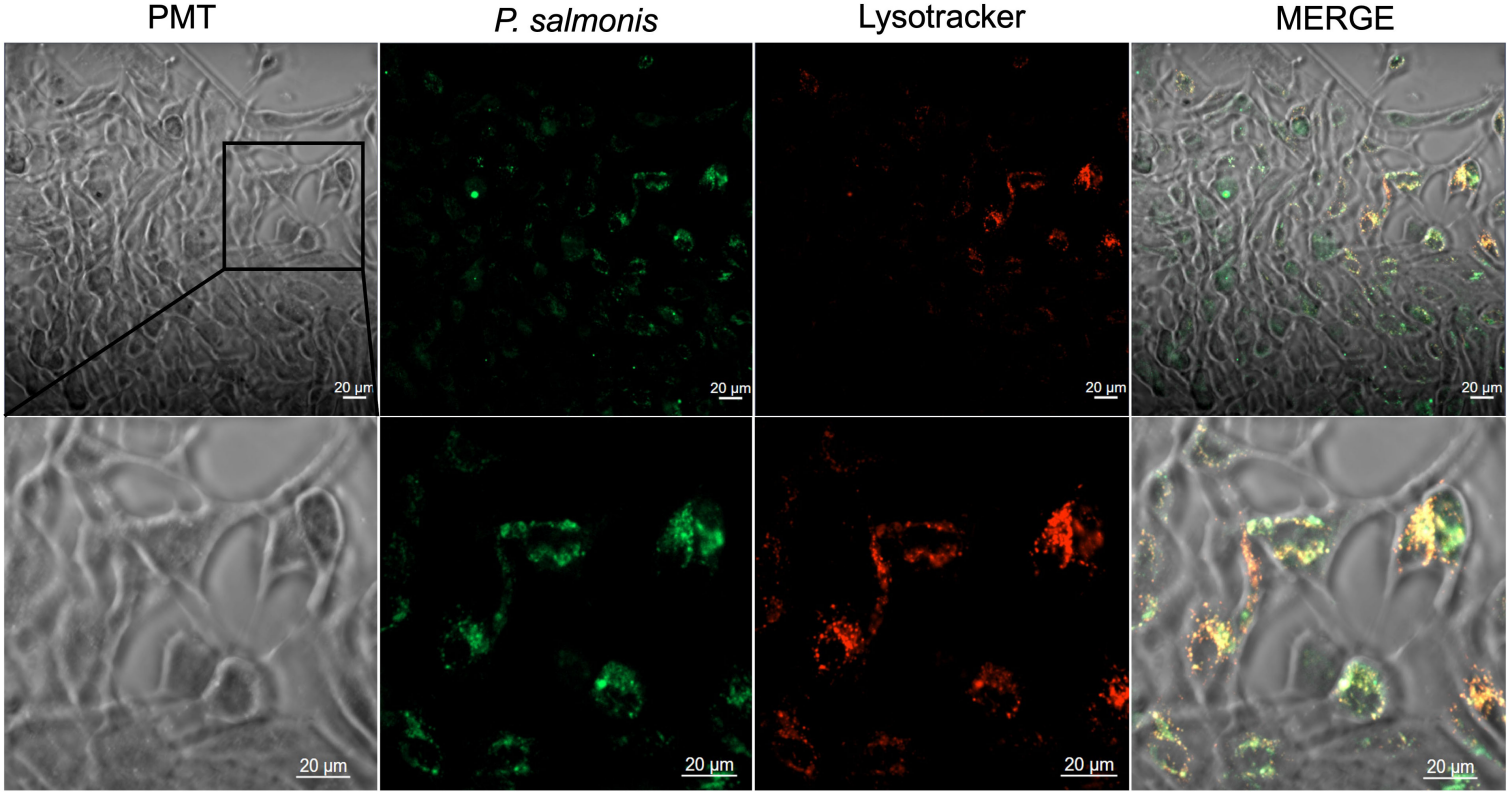
Confocal microscopy of dorsal fin-derived cells from Atlantic salmon infected with inactivated *Piscirickettsia salmonis.* Primary dorsal fin cells were infected for 24 h with SYTO 9-labeled *P. salmonis* (green) and stained with Lysotracker Red to visualize lysosomal compartments. Merged images show clear colocalization (yellow/orange signal) between bacterial and lysosomal fluorescence. Images were acquired using LD A-Plan 40X/0.55 Ph1.

To assess whether *P. salmonis* infects dorsal fin-derived cells from Atlantic salmon, confocal microscopy was performed using live bacteria labeled with SYTO 9 (green) and lysotracker Red as a lysosomal marker (n=3). After 24 hours of infection, the bacteria were distributed throughout the dorsal fin cells, with staining overlapping lysosomes, suggesting that the bacteria trafficked toward the antigen-processing compartments (**Figure 5A**; **Supplementary Figure 4**). The autofluorescence controls shown in **Figure 5B** indicated that fluorescence emission was not detected in the fin cells. An orthogonal view of the infected cells confirmed the localization of *P. salmonis* within the cells (**Figure 5C**). Comparable results were obtained when dead bacteria were used for infection (**Figure 6**). This finding indicates that uptake is independent of bacterial metabolism, invasion factors, or viability, and suggests that dorsal fin cells passively participate in bacterial uptake. The *P. salmonis* uptake, co-localization with lysosomes, and the presence of MHC-I in the fin cells suggest that these cells process and present bacterial antigens for evaluating T-cell-mediated cytotoxicity in an autologous *ex vivo* model.

### 3.3 Evaluation of cytotoxic T cell responses against *P. salmonis*

**Figure 7:**
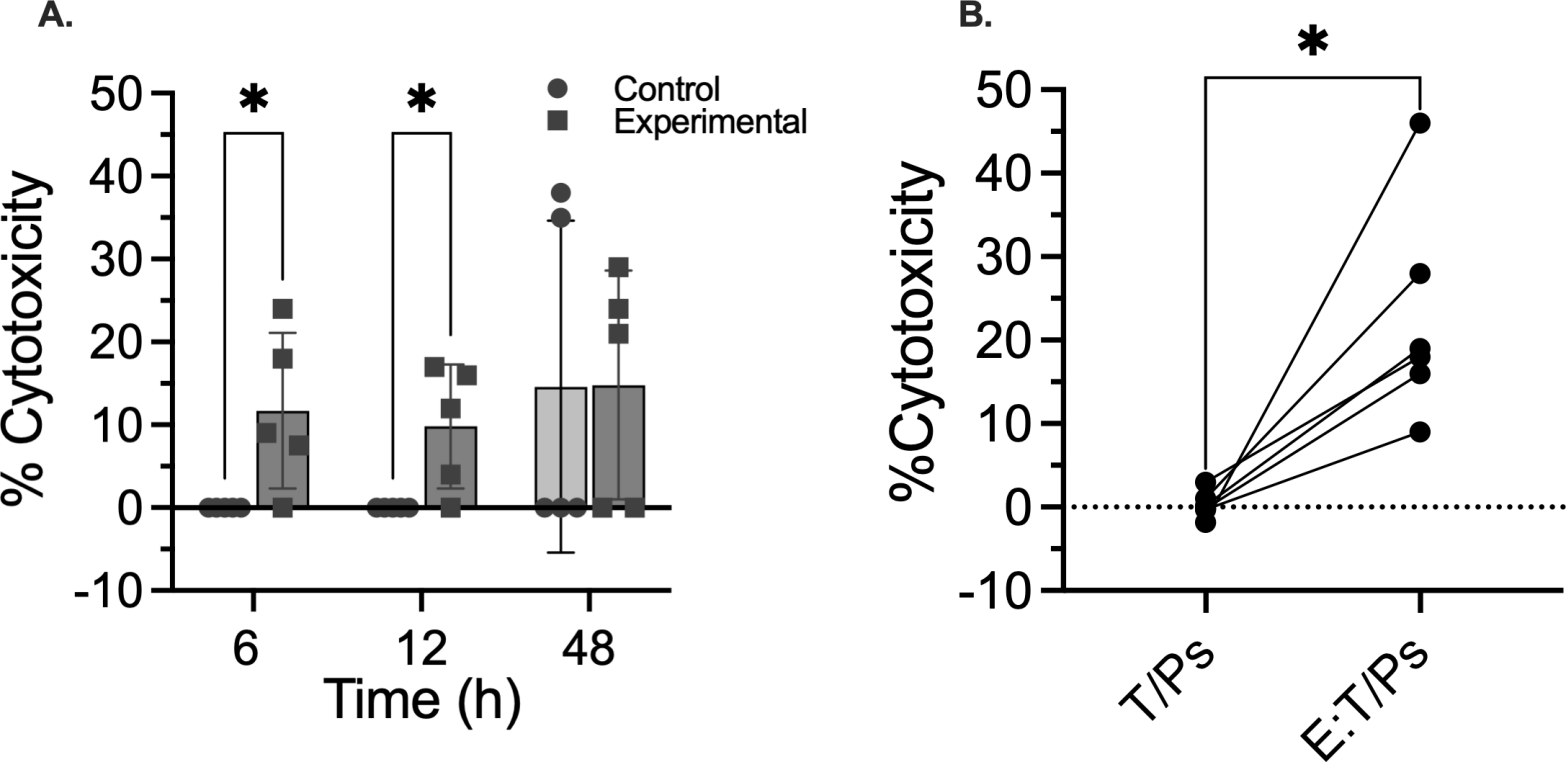
Cytotoxic activity of activated PBL against *P. salmonis*-infected fin-derived cells. **(A)** *P. salmonis*-infected fin cells were co-cultured with isogenic sensitized PBL of *P. salmonis*-infected rainbow trout for 6, 12, or 48 horas at 16 °C. Cellular medium was collected at each time point, and lactate dehydrogenase (LDH) release was quantified using the CyQUANT™ LDH Cytotoxicity Assay Kit. Data represent mean ± standard error from n=5 independent biological replicates per time point. **(B)** Cytotoxic activity of total sensitized peripheral blood leukocytes (PBL) against fin target cells from *P. salmonis*-infected Atlantic salmon exposed to live *P. salmonis* (n=6). Statistical significance was assessed using a paired t-test (*p < 0.05). T/Ps indicates target cells infected with *P. salmonis*, while E:T/Ps denotes effector cells incubated with target cells infected with *P. salmonis*.

The measurement of the cytotoxic capacity of fish T lymphocytes against *P. salmonis*-infected isogenic fin cells required standardizing the LDH enzymatic reaction conditions, optimizing the effector-to-target (E:T) ratio, adjusting co-incubation conditions (time and temperature), and selecting appropriate controls. The E:T incubation was assessed at 18 °C, which is the optimal temperature for salmonid cell growth, using an E:T ratio of 10:1. For LDH detection, two different temperatures were utilized: 18 °C, the temperature for cell culture, and 37 °C, the recommended temperature for the LDH assay, with 37 °C being the best temperature for analysis. Regarding the incubation time for the effector and target cells, **Figure 7A** shows that a 6-12-hour incubation at 18 °C was optimal for assays 1 The spontaneous lysis of target cells in the control group at 48 hours of incubation was so extensive (**Figure 7A**) that it was not possible to perform the cytotoxicity assay under these conditions. This standardization condition was also applicable to the assays performed with Atlantic salmon. The specific cytotoxic capacity of *P. salmonis*-sensitized T lymphocytes (effector cells) from Atlantic salmon was assessed by measuring LDH release from infected fin cells (target cells) after 12 hours E:T co-incubation at 18 °C. Control groups were uninfected fin cells (spontaneous lysis), fin cells infected with *P. salmonis* without the addition of effector cells (lysis induced by infection), and uninfected fin cells co-cultured with sensitized peripheral blood lymphocytes (non-specific lysis). Results showed that antigen-specific lysis rates ranged from 5% to 46% (**Figure 7B**, T/Ps:E), while infection-induced lysis of fin cells (**Figure 7B**, T/Ps) and non-specific lysis (not shown) were negligible. At this stage, results demonstrated the presence of antigen-specific cytotoxic leukocytes in *P. salmonis*-infected fish.

### 3.4 CD8+ T cells as cytotoxic lymphocytes

**Figure 8:**
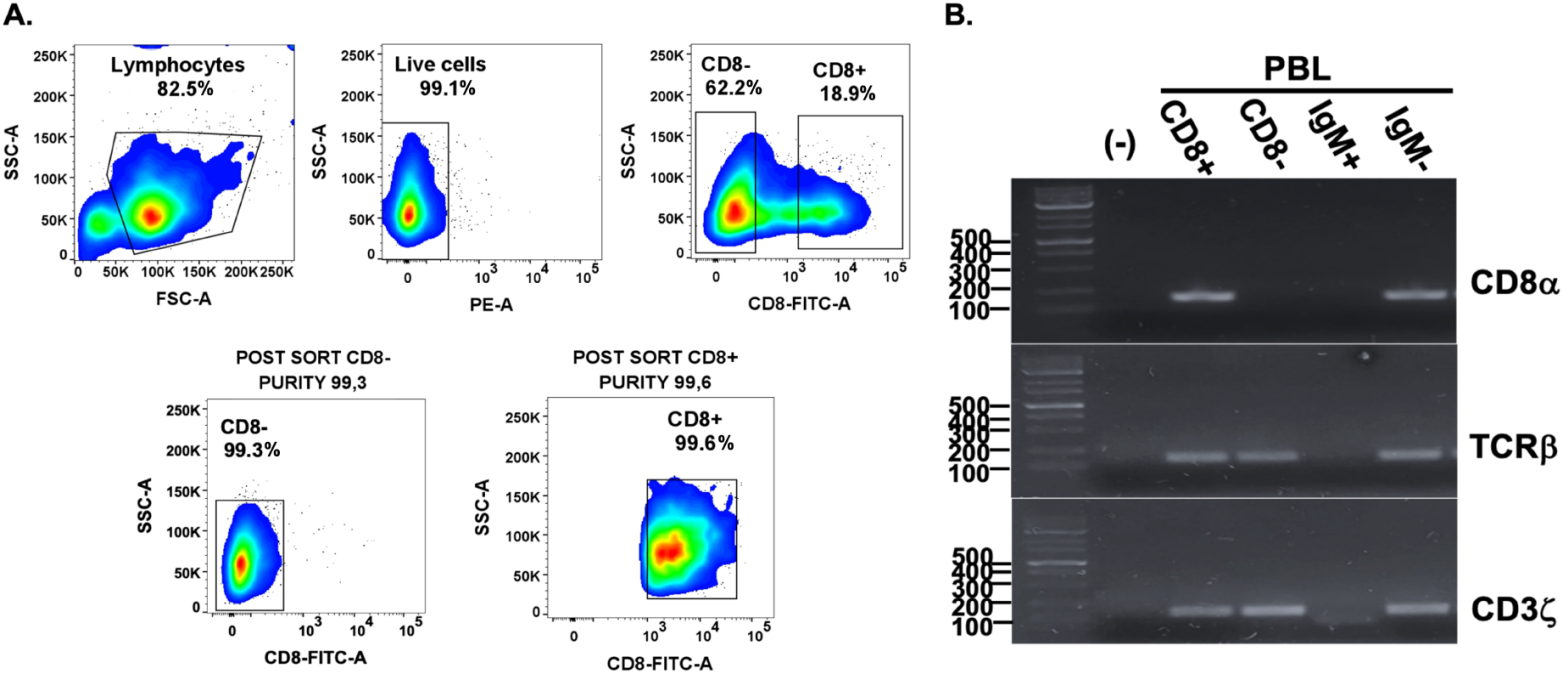
Pre− and post−sorting analysis of CD8-lymphocytes and RT-PCR analysis of sorted cells from Atlantic salmon PBL. **(A)** Peripheral blood leukocytes obtained from infected salmon were isolated and gated on lymphocytes based on their characteristic FSC^low^/SSC^low^ profile. PI positive cells were excluded, and PI unstained cells were considered viable. Within the lymphocyte gate, CD8^+^ cells were excluded, and the remaining CD8− population was selected for sorting collection. The lower panel shows the post−sort analysis, confirming successful enrichment of CD8− lymphocytes and post-sort profile of the CD8+ fraction. Numbers show the percentage of gated cells regarding total counts **(B)** RT-PCR analysis of sorted PBL population. CD8α transcripts were detected exclusively in the CD8α+ fraction, while TCRβ and CD3ζ transcripts were present in CD8α+ but absent in CD8- and IgM+ fractions.

**Figure 9:**
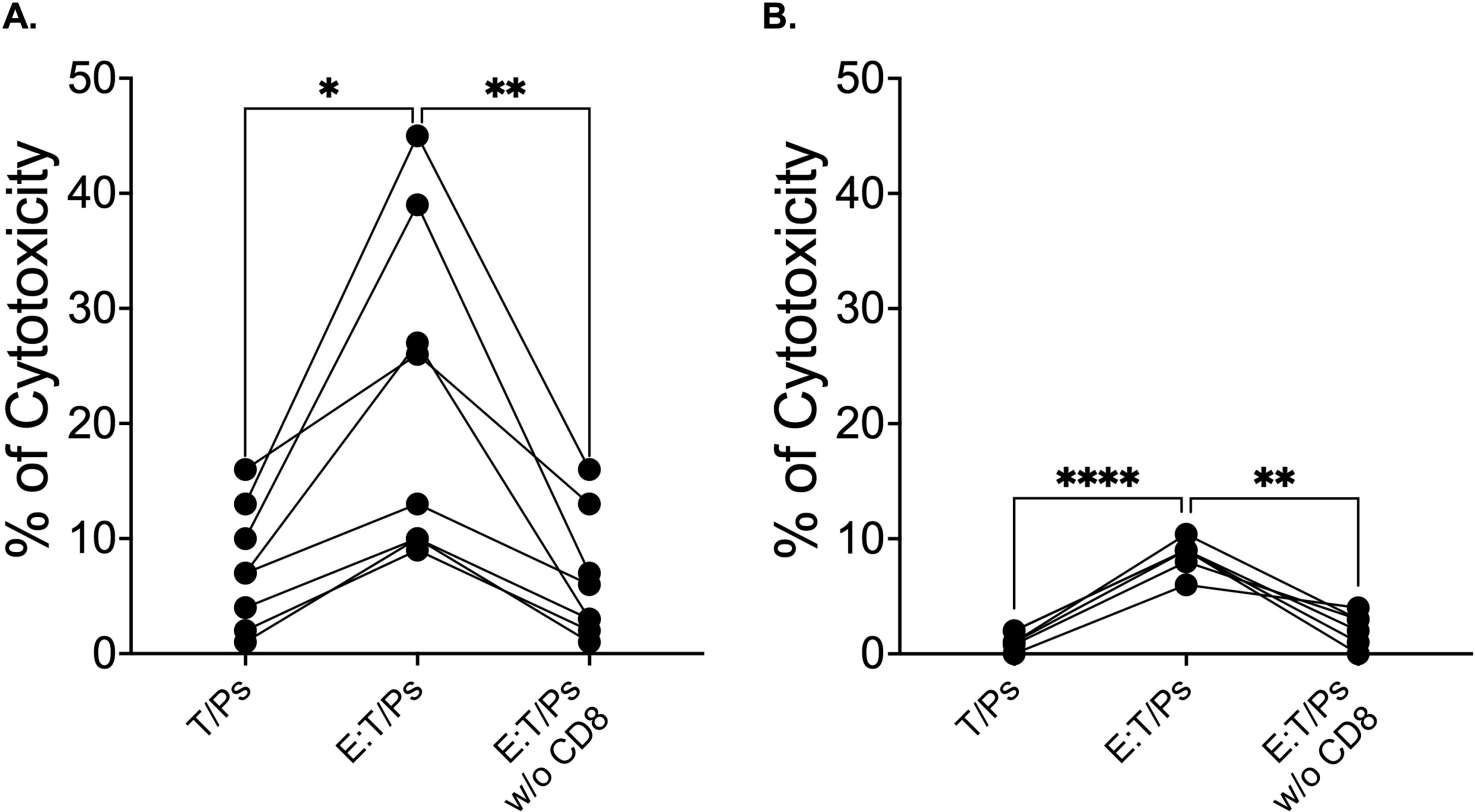
*Ex vivo* cytotoxicity assays using dorsal fin-derived target cells from Atlantic salmon with CD8 lymphocyte depletion. **(A)** Cytotoxic assay with effector cells depleted of CD8+ lymphocytes and exposed to fin cells infected with live *P. salmonis* (n=7). **(B)** Cytotoxic assay with effector cells depleted of CD8+ lymphocytes and exposed to fin cells infected with inactivated *P. salmonis* (n=6). T/Ps refers to target cells infected with *P. salmonis*. E:T/Ps effector cells incubated with target cells infected with *P. salmonis*. E:T/Ps w/o CD8 refers to the same assay, but with effectors lacking CD8+ cells. Statistical significance was assessed using one-way ANOVA Tukey's multiple comparisons test. * p < 0.05 ; **p < 0.01; ****p < 0.0001.

To determine if the cytotoxic T cells of Atlantic salmon responding against *P. salmonis* were CD8+ T cells, sensitized leukocytes obtained from infected salmon were labeled with a monoclonal anti-CD8α antibody. Then, leukocytes depleted of CD8+ cells were recovered after cell sorting, with more than 99% purity (**Figure 8A**). CD8+ cells isolated by cell sorting were processed to examine the expression of a set of T lymphocyte-specific genes using RT-PCR. Transcript analysis results (**Figure 8B**) showed that CD8+ cells isolated from PBL express CD8α, CD3ζ, and TCRβ, indicating that they are CD8+ T lymphocytes. Using CD8-depleted sensitized leukocytes and the non-depleted sensitized leukocytes, we tested antigen-specific cytotoxicity against *P. salmonis*. Sensitized leukocytes from all fish tested produced LDH release in a range of 9-45% on target cells infected with *P. salmonis* (**Figure 9A**). In contrast, the depletion of CD8α+ cells resulted in a reduction in cytotoxicity that reached the baseline levels (**Figure 9A**). To determine whether the viability of *P. salmonis* influences the antigen presentation ability of the target cells (fin cells), primary dorsal fin cell cultures from Atlantic salmon were incubated with heat-inactivated *P. salmonis*. Under these conditions, we also observed LDH release from the target cells, but it ranged from 6-10% (**Figure 9B**). As before, the depletion of CD8α+ cells resulted in a reduction in cytotoxicity to the basal levels.

## 4 Discussion

This study presents functional evidence that antigen-specific CD8α+ cells mediate cytotoxicity against *P. salmonis*-exposed syngeneic cells in Atlantic salmon. *P. salmonis* is a facultative intracellular pathogen that survives and replicates within fish cells, and the clearance of such organisms generally requires immune mechanisms targeting infected host cells (46). The finding that fish-sensitized leukocytes mediate antigen-specific lysis of autologous, intracellularly exposed target cells supports a model in which CTL-like effector activities contribute to limiting intracellular bacterial reservoirs in Atlantic salmon.

To investigate antigen-specific T cell cytotoxicity in Atlantic salmon, the challenge of the absence of clonal or inbred salmon was first addressed. Short-term autologous cultures were established from fin samples of tagged fish as target (36, 47), and haplotype-matched effector leukocytes from the same tagged fish were used for cytotoxicity assays. Because fin cultures comprise a heterogeneous population of uncharacterized adherent cell types, the ability of *P. salmonis* to infect fin-derived cells *in vitro* was examined to generate infected target cells. *P. salmonis* is classically reported to infect macrophages and monocytes (e.g., head−kidney macrophages and cell lines such as SHK−1 and RTS11) (48). Our results confirmed that the bacteria were internalized by salmon fin cells, which may contain resident phagocytic cells, as well as other susceptible epithelial or stromal cell types that *P. salmonis* can invade. Confocal microscopy demonstrated colocalization of *P. salmonis* with lysosomal compartments in fin cells, indicating active trafficking toward degradative pathways (49), which may contribute to reducing bacterial burden in salmon macrophages (50).

The presence of CD80 and CD86, which are essential costimulatory molecules on antigen-presenting cells, as well as CD4, a marker found on human macrophages involved in differentiation, activation, cytokine release, and migration (51, 52), and MHC-I, a critical component for bacterial antigen presentation, indicates that fin cells can present *P. salmonis* antigens for T cell recognition. Flow cytometry analysis revealed the surface expression of MHC-I, CD80/86, and CD4-1, supporting their biological functions. However, quantitative data on the abundance of these molecules in salmon fin cells require validation with additional samples and more comprehensive analyses. The co-localization of lysosomes and infected bacteria in the salmon fin cells suggests that lysosomal degradation may occur and intersect with MHC-I antigen processing (49), allowing cells to present bacterial peptides to CD8+ T cells. This process is well documented in mammals and is increasingly recognized in teleost mucosal and stromal tissues (53–55). Collectively, these biological features support the use of fin-derived cultures as target cells in cytotoxicity assays.

The main and novel finding of this study is that infection of Atlantic salmon by *P. salmonis* induces antigen-specific cytotoxicity as a defense mechanism against this intracellular bacterium. Since this response was markedly diminished following CD8+ cell depletion and these cells express T cell markers, the results strongly indicate that CD8+ T cells are key mediators of cytotoxicity in *P. salmonis-*infected cells. Cytotoxic mechanisms that can implicate perforin/granzyme pathways (56) remain to be identified. Thus, assessment of granzyme, perforin, and IFN-γ expression in sorted CD8+ T cells following co-culture with infected fin-derived cells, microscopic examination of subcellular compartments in effector and target cells, and live-cell microscopy to visualize cytotoxic dynamics are included in a follow-up study designed to determine whether cytotoxicity is granule-dependent and associated with IFN-γ–mediated pathways.

Some transcriptomic and gene-expression studies in Atlantic salmon infected with *P. salmonis* or following vaccination have reported upregulation of genes associated with cell-mediated cytotoxicity, including *cd8*α, cd8β, cd4, and *ifn-*γ (57, 58). Those molecular signatures have been interpreted as indicative of cell-mediated immune activation, but direct functional evidence of CTL-mediated killing of infected somatic cells has been limited. Our functional assays, therefore, extend prior molecular observations by showing antigen-specific lytic activity attributable to Atlantic salmon CD8α+ T cells, bridging the gap between gene-expression correlation and effector function. In other teleost fishes, these findings align with previous reports showing that intracellular pathogens induce cytotoxic T lymphocyte (CTL) responses. For instance, studies in ginbuna crucian carp have shown the development of virus-specific CD8+ CTLs against CHNV (24, 26). Similarly, in rainbow trout, peripheral blood leukocytes (PBL) from VHSV-infected or G-protein–vaccinated individuals killed MHC I–matched VHSV-infected cells (28, 29). This pattern is also observed in orange-spotted grouper, where CD8α+ PBL exhibit MHC I–restricted cytotoxicity against NNV (18). Notably, the current results are also consistent with evidence that cell-mediated immunity is essential for the clearance of *Edwardsiella tarda* in ginbuna crucian carp, as the elimination of this intracellular bacterium is associated with increased CTL activity and higher numbers of CD8α+ cells (59). To date, aside from the present study, only the Yamasaki study has reported a CTL immune response against intracellular bacteria in fish. Collectively, these studies and the current findings underscore the evolutionary conservation of CTL effector mechanisms against intracellular microorganisms across teleost species.

From a vaccinology perspective, these findings accentuate the need for strategies that potentiate CTL responses against *P. salmonis*, rather than relying solely on antibody-mediated protection. Current subunit and live-attenuated vaccines against *P. salmonis* induce limited cellular immunity (60), and reverse vaccinology approaches targeting T-cell epitopes are emerging as promising alternatives (61). Understanding how CD8+ T cells interact with infected epithelial and fibroblast-like cells in salmonids may inform the design of next-generation vaccines that enhance antigen-specific cytotoxicity and long-term protection (14, 62).

The observation that heat-inactivated bacteria can be recognized by CD8+ T cells indicates that bacterial metabolism is not strictly required for phagocytosis, antigen processing, MHC loading, and presentation in the target cells. However, the reduced cytotoxicity observed compared with targets exposed to live bacteria supports the hypothesis that *P. salmonis* or other pathogen-associated signals enhance cellular uptake, antigen processing, or presentation. These findings have practical implications for vaccine strategies that utilize inactivated antigens (63). This underscores the importance of identifying molecules that provide pathogen-associated molecular pattern (PAMP)-driven stimulation to achieve cell-mediated immunity levels comparable to those induced by live bacterial activation. Mucocutaneous tissues, such as skin, gills, and fins, serve as interfaces with the aquatic environment and harbor resident macrophage-like cells and lymphocytes. The ability of the fin cells to participate in bacterial uptake and antigen processing suggests they may contribute to local bacterial containment and the restimulation (and maybe priming) of effector T cells *in situ*. Such local cellular response could be significant in aquaculture, where skin breaches, handling stress, and high stocking densities enhance localized pathogen exposure and transmission.

In summary, this study provides the first evidence that infection of Atlantic salmon *with P. salmonis* induces antigen-specific cytotoxic activity mediated by CD8+ T cells. These results underscore the significance of cell-mediated immunity in host defense against intracellular pathogens. For the future, establish MHC I–restricted cytotoxicity in *P. salmonis* CTL response in Atlantic salmon remains an open area of study. A single expressed MHC class I locus exists in Atlantic salmon, *Sasa*-UBA but the MHC class I genes showed many alleles differing by distinct patterns of amino acid substitutions (64, 65). Thus, understanding MHC restriction will require the identification of the haplotypes of Atlantic salmon used in the experiments and testing peptide-specific, MHC I–restricted killing using haplotype-matched and -mismatched targets. In addition, elucidate the role of CD8+ cytotoxic T cells in controlling Piscirickettsiosis will be critical to improve vaccine design for salmon against *P. salmonis*, incorporating cellular immunity induction as endpoints.

## Data Availability

The raw data supporting the conclusions of this article will be made available by the authors, without undue reservation.
