# Correction: *Piscirickettsia salmonis* elicited an antigen-specific cytotoxic response dependent on CD8+ T cells in Atlantic salmon

**DOI:** 10.3389/fimmu.2026.1849843

**Published:** 2026-05-18

**Authors:** Felipe Barraza-Rojas, Valentina Wong-Benito, Daniel Valdés-Henriquez, Kevin Maisey, Brian Dixon, Mónica Imarai

**Affiliations:** 1 Centro de Biotecnología Acuícola, Departamento de Biología, Facultad de Química y Biología, Universidad de Santiago de Chile, Santiago, Chile; 2 Department of Biology, University of Waterloo, Waterloo, ON, Canada; 3 Laboratorio de Microscopía Fotónica Avanzada, Departamento de Biología, Facultad de Química y Biología, Universidad de Santiago de Chile, Santiago, Chile; 4 Laboratorio de Inmunología Comparativa, Centro de Biotecnología Acuícola, Universidad de Santiago de Chile, Santiago, Chile

**Keywords:** antigen-specific cytotoxicity, Atlantic salmon, CD^8^+ T cell, cell-mediated immunity, cytotoxic T lymphocyte, *Piscirickettsia salmonis*, teleost immunity

Equation in **Methods** section number 2.12 was erroneously given as:

“% of cytotoxicity = ^100^ x (LDH release in Experimental - Spontaneous LDH release).

Total LDH release - Spontaneous LDH release.”

The correct equation is:


$$“\%\;of\;cytotoxicity=100\text{ x}\frac{\left(\text{LDH release in Experimental}-\text{Spontaneous LDH release}\right)}{(\text{Total LDH release}-\text{Spontaneous LDH release})”}$$

The original version of this article has been updated.

