# Supplementary material for: Piscirickettsia salmonis elicited an antigen-specific cytotoxic response dependent on CD8+ T cells in Atlantic salmon
Source: Front Immunol. 2026 Apr 1;17:1803394. doi: 10.3389/fimmu.2026.1803394 (PMC13079172; doi:10.3389/fimmu.2026.1803394)
Supplement: Supplementary file 1 [file DataSheet1.pdf]

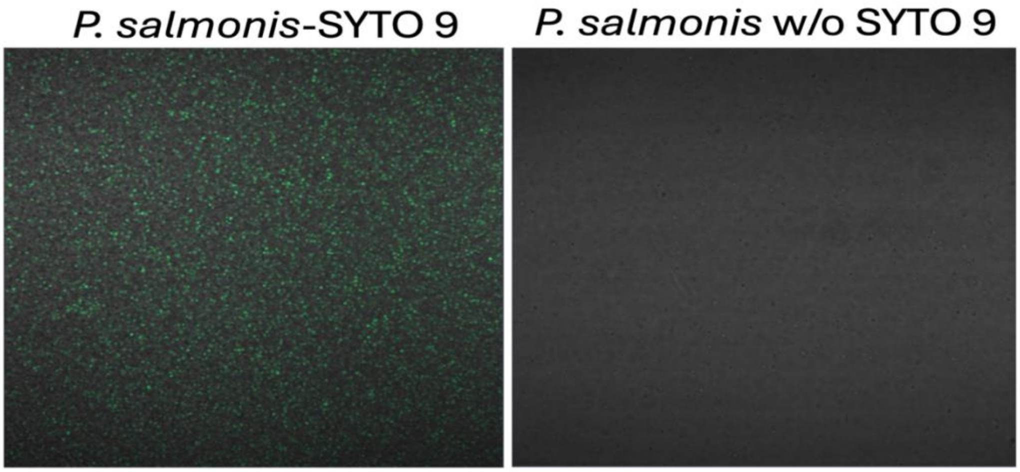

Supplementary Figure 1. Confocal microscopy of SYTO 9-labeled *P. salmonis*. Confocal micrographs showing *P. salmonis* stained with Syto 9 (left) and an unstained control (right). SYTO 9-labeled bacteria exhibited strong green fluorescence, while the unstained sample showed no detectable signal. Images were acquired using LD A-Plan 40X/0.55 Ph1.

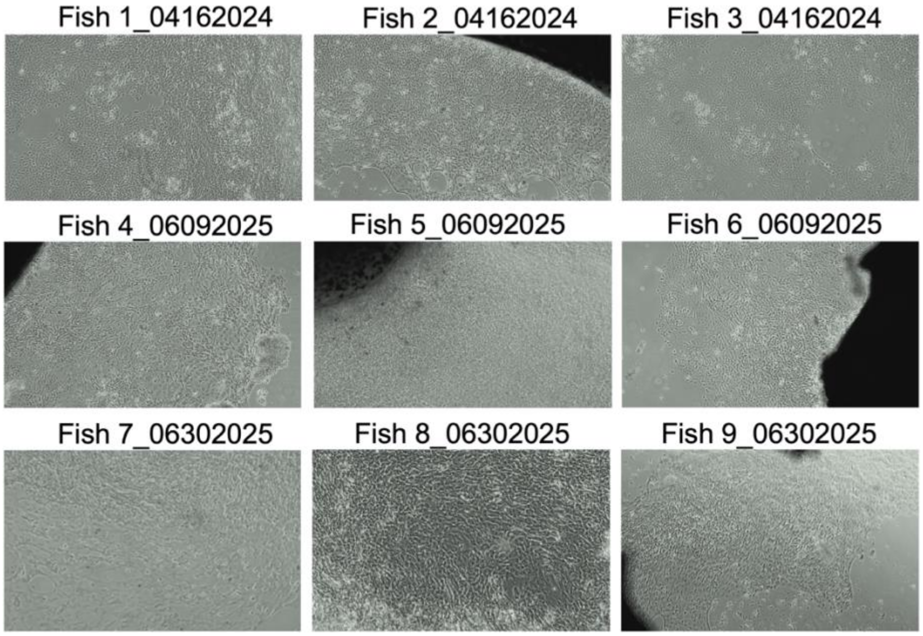

Supplementary Figure 2. Target cells derived from the dorsal fin of Atlantic salmon. Optical microscopy images show primary cell cultures from the dorsal fin after 7 days of incubation. The cultures exhibit a heterogeneous population consisting of small, rounded epithelial-like cells and larger, elongated fibroblast-like cells distributed across the culture surface. (n=9)

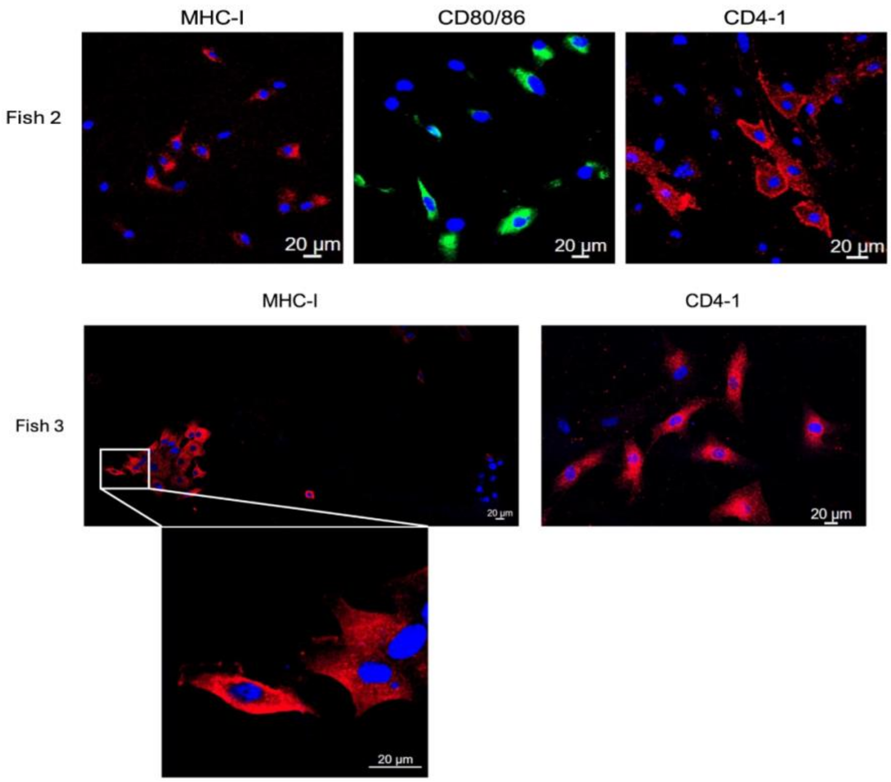

Supplementary Figure 3. Expression of MHC-I, CD80/86, and CD4-1 in fin-derived cells analyzed by confocal microscopy and detection of MHC-I gene expression by conventional PCR. **(A)** Confocal immunofluorescence images showing the expression of MHC-I (red), CD80/86 (green), and CD4-1 (red) in fin-derived cells. Nuclei were stained with DAPI (blue). The upper panel shows general views; the lower panel shows higher magnification of representative cells. Images were acquired using alpha Plan-Apochromat 63x/1.46 Oil M27. Upper figures (Fish 2). Lower panel (Fish 3).

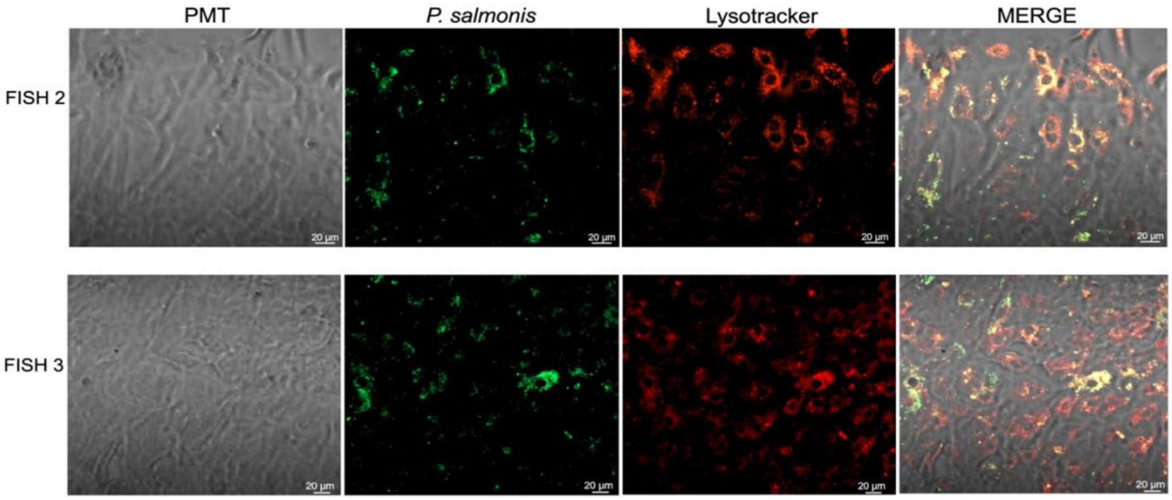

Supplementary Figure 4. Confocal Imaging of dorsal fin-derived cells from Atlantic salmon infected with *P. salmonis*. (A) Primary dorsal fin cells were infected for 24 h with SYTO 9-labeled *P. salmonis* (green) and stained with Lysotracker Red to visualize lysosomal compartments. Merged images show clear colocalization (yellow/orange signal) between bacterial and lysosomal fluorescence. Images were acquired using LD A-Plan 40X/0.55 Ph 1. Upper figures (Fish 2). Lower panel (Fish 3).
